# Enhancing Chinese EFL Students' Grit: The Impact of Teacher Stroke and Teacher-Student Rapport

**DOI:** 10.3389/fpsyg.2021.823280

**Published:** 2022-01-21

**Authors:** Lingjie Yuan

**Affiliations:** School of Foreign Languages, Xinxiang Medical University, Xinxiang, China

**Keywords:** students' grit, teacher stroke, teacher-student rapport, positive teacher interpersonal behaviors, language learning

## Abstract

Due to the important role that students' grit plays in the effectiveness of their success in the educational system in which they are engaged, the current study scrutinized whether some factors regarding teachers such as their stroke and rapport can affect the learners' grit in one hand and on the other hand whether these factors can predict the learners' grit or not. To this end, a group of 316 Chinese university English as a Foreign Language (EFL) learners from more than 30 cities from nine provinces of China were asked to fill out the three scales, namely, teacher stroke, student rapport, and students' grit questionnaires. The foremost findings of the study, gained through running regression, indicate that there are positive relations between these variables as they affect learners' grit and also both variables were the predictors of grit, while teacher stroke was a better predictor, uniquely clarifying 45.5% of the grit's variance and teacher-student rapport similarly showed to be a predictor of grit, distinctively clarifying 4.6% of its variance. Accordingly, based on these findings, it can be concluded that both of these factors, as instances of positive teacher interpersonal behaviors, develop learners' grit in language learning. In addition, this study can provide further implications and recommendations for language teaching team members in academic circumstances.

## Introduction

Grit, as a relatively new conception in the education and positive psychology (PP) paradigm, encompasses the theories of passion and persistence (MacIntyre et al., 2019; Mattick et al., 2021; Wang et al., 2021), that has received attention in further various parts, comprising professional, medicinal facilities, and education (Sudina et al., 2021). Grit is said to be a fundamental component in the process of language teaching (Keegan, 2017) and it is known in the series of works as a useful topic for those who are likely to thrive in education since it has been woven with better commitment and academic competence (Reed and Jeremiah, 2017; Hodge et al., 2018). In addition, it was suggested as an important psychosocial element in predicting success in scholastic performance (Li et al., 2018). Grit has been suggested to have a vital function in helping learners make the best effort to improve and cultivate their language skills regardless of the hardships they are confronted with (Keegan, 2017). Furthermore, grit is a self-directed and non-academic developmental trait that consists of two central components: (a) perseverance in effort and (b) persistence of interests (Duckworth and Quinn, 2009). While the earlier is the effective hunt for goals in the face of setbacks, the latter involves accountability and vigor exerted for a goal. A combination of these two traits has been expected for several years, as grit has the plan of compensating for the long-discussed association and partnership between the vigor and perseverance required to impact lasting goals (Duckworth, 2016).

Grit has a close connection with the educational progression and scholastic accomplishment that has been frequently and constantly mentioned in various general settings (Ha et al., 2015; Hwang et al., 2017; Choi, 2018). However, since grit is still a moderately new notion in this realm, many of the current inquiries have emphasized examining its aspects and interactions with other factors. Investigations that have directly focused on improving grit are still rare. The development of instructive mediations that foster grit has turned into a significant part of the schedule. Duckworth (2016) proposed that the internal circumstances for the improvement and progress of grit are the well-being, behaviors, objectives, and hopes of the individual, as well as guardians, educators, learning experiences, and cultural settings.

Educators have been perceived to have an impact on the effectiveness of educational systems and student performance, as a key stakeholder in the teaching system. The significant function of educators in making various school decisions related to students has led to the inference that the behavioral, psychological, and teaching traits of teachers deserve due consideration and investigation (Burroughs et al., 2019; Derakhshan et al., 2020). Educators are often the primary source of educational input in the school, creating the situations for teacher-student and student-student associations in the classroom situation. Moreover, they are accountable for developing a positive teaching environment and long-term impact on the educational results of learners (Burroughs et al., 2019). As stated by Li (2006), any incorporated emphasis on education cannot be separated from highlighting the educator-learner rapport. It is not just because educator-learner rapport is a relevant measurement in instruction, but because teaching and education should be completed inside the educator-learner rapport.

In addition, the teacher-student relationship generates the emotive bond of the student, and this connection pays off in language teaching, where teachers and students are in constant communication, creating friendlier associations and participating in social communication to improve the development of the student's language skills (Pishghadam et al., 2019; Fathi et al., 2021). Educators and learners are mutually accountable for the influential execution of the learning and teaching developments; therefore, they need to work together to make positive learning conditions (Gabry's-Barker and Gałajda, 2016). Teacher-student relations are viewed among the noteworthy features assumed to support the learner in the classroom (Wang and Derakhshan, 2021; Xie and Derakhshan, 2021). Learners become knowledgeable and skilled as they engage in classroom activities, enhance societal skills, and begin to establish a personal identity as they employ considerable time there. Educational experiences that occur in the classroom may also directly impact learners' behavior and socialization skills as they progress through the academic process (Skinner and Pitzer, 2012). The activities and communication that take place outside and inside the classroom have a significant impact on the performance and enthusiasm of students who are spending most of their time in that setting (Derakhshan, 2021; Pishghadam et al., 2021).

As stated by Hughes (2012), increasing educators' support for learners has a good effect on reducing depressive symptoms and obtaining greater self-confidence, and they can enhance behavior in a social context. Years of studies in the field of educational communication have confirmed the claim that effective communicative behavior by teachers can lead to favorable learner-related scholastic results (Pishghadam et al., 2021). Educators provide learners with effective psychological support through interaction with them, resulting in both emotional stability and academic success (Pekrun and Schutz, 2007). Some qualities are evidence of a strong educator-learner rapport such as sympathy, concern, engagement, optimism, and respect and among the effective characteristics of educators examined today, including support, enthusiasm, scholastic immediacy, honesty, clarity, encouragement, and appreciation (Frisby, 2019). Teacher-student rapport is a necessary part of achieving any type of education as creating and maintaining a positive relationship between a professor and a learner is an exceptionally difficult task for some accomplished educators (Strachan, 2020), As specified by Frisby et al. (2016), comparatively, rapport is less scrutinized than other issues associated with academic communication. It is still the most important component of the learning process because it is an inescapable aspect of instruction, so it is the foundation for learners' learning.

Educator stroke is defined as any gesture taken by an educator in acknowledgment of the individuals around him/her (Peng and Woodrow, 2010), which is an essential feature of the theory of Transactional Analysis (TA). It is used in the academic setting to describe social interactions between learners and their educators. Learners can be stroked by educators by mentioning them by name, giving positive feedback, engaging, keeping in touch with the educator continuously, nodding, maintaining eye contact, encouraging class discussion, and laughing (Irajzad et al., 2017). Pishghadam et al. (2015) broadening the concept of the stroke to include “kind,” “helpful,” and “positive behavior,” considered it one of the essential parts of educator support. Those who are engaged in this type of teaching behavior strive to establish and maintain a strong rapport with their learners (Zheng, 2021). Since stroke is related to increased motivation in learning, it can be concluded that if the teacher is attentive to all learners, involves them in class discussions, praises their performance in front of others, as the strokees are more motivated, they try harder to learn, and this increases their performance level (Pishghadam and Khajavy, 2014). Also, the rapport between learners and educators is of crucial importance given that both are equally responsible for ensuring a fruitful academic and teaching process (Delos Reyes and Torio, 2020; Meng, 2021).

Some studies have currently carried out about teacher stroke (e.g., Irajzad et al., 2017; Noorbakhsh et al., 2018) and the noteworthy function of teacher-student rapport is guaranteed on issues such as commitment, accomplishment, passion, and self-confidence (Derakhshan et al., 2019; Pishghadam et al., 2019; Havik and Westergård, 2020; Derakhshan, 2021). However, grounded on the researcher's knowledge, the presentation of an empirical study on positive interpersonal behaviors, namely stroke and teacher-student rapport in the EFL context and its relation to learners' grit has been overlooked. Regarding this gap, the present research makes efforts to explore not only their relation but also their predictive role of them upon learners' grit. To this end, the following questions were formulated in the current research.

Q_1_. Is there any significant relationship between Chinese EFL students' grit, teacher stroke, and teacher-student rapport?
Q_2_. Does teacher stroke predict Chinese EFL students' grit in the classroom?
Q_3_. Does teacher-student rapport predict Chinese EFL students' grit in the classroom?

## Review of the Literature

### Students' Grit

Grit is deemed as a kind of personality that reflects the amount of vigor that people spend in developing ambitious goals that will contribute to their development (Duckworth, 2016). Furthermore, it is one of the main predictors of a student's scholastic achievement; in other words, students who work hard are fascinated by what they perform and are more likely to deal with language learning barriers, thereby leading to enhanced performance (Dweck et al., 2014). According to Duckworth and Yeager (2015), grit is a personal quality common to leaders, and a substantial precursor to success and immensity in any direction regardless of knowledge or experience. Learner achievement, as well as philosophical beliefs such as optimism and enthusiasm, was greatly influenced by grit, as a psychological personality trait (Datu et al., 2019). Furthermore, Ryan and Deci (2000) suggested that learners with grit are stimulated by a personal interest in the process of learning, and those with personal goals have a greater sense of motivation, self-assurance, and excitement, which ultimately results in improved performance, persistence, well-being, and creativity. As stated by Duckworth et al. (2007), grit is the competence to continue learning after difficulties, and research indicates that grit has a significant stimulus on perseverance, discipline, and management, along with a positive mental attitude in attaining goals (Reed and Jeremiah, 2017). Continuous interest in a task and a determination to succeed are two components of grit, which are psychological and permanent mental attitudes. The presence of these features can provide different outcomes among students with concurrent academic and intellectual abilities (Duckworth et al., 2007). To achieve the growth of social expression in all other dimensions of life and can be seen as a social expression or an ethical value, grit is a focal and indispensable matter (Sariçam et al., 2015). Research has shown that it is related to achievement, self-efficacy, self-control, metacognition, hopelessness, and stress (Lee, 2020; Fathi et al., 2021). Grit implies participation in a superordinate goal that is at the maximum level of a pyramid, while the lower-level goals are connected and anticipate moving toward the superordinate goal (Duckworth and Gross, 2014).

### Teacher Stroke

Stroke is used primarily as an indication of educator feedback and praise in scholastic psychology (Hattie and Timperley, 2007). It is worth mentioning that various studies are available on the two notions just mentioned (Pishghadam et al., 2015). A stroke-rich classroom environment encourages L2 students to perform better and if the L2 students are successful, the teachers are also considered successful. Educators (strokers) can stroke learners (strokees) in different ways, like how to call the names of the students, let them express themselves, provide adequate feedback, and promote students in different ways (Pishghadam and Khajavy, 2014). Educator strokes are effective tools to strengthen the academic motivation of L2 students and to enhance their behavior (Akin-Little et al., 2004). Due to the emphasis on caring for students, stroke is meticulously and thoroughly associated with feedback, particularly one that arranges for positive and negative assessments of students (Hattie and Timperley, 2007). Strokes are divided into verbal and non-verbal. A verbal stroke is a series of phrases that are used as a kind of recognition for another, while a non-verbal stroke can range from a simple physical touch to a smile or even an affirmative assent or dismissal (Stewart and Joines, 2008).

### Teacher and Student Rapport

The word “rapport” lexically describes a friendly connection in which one gets along very well (Allen et al., 2013). In the academic setting, it is the relationship that learners have with each other and with educators (Ahmad, 2018). In other words, maintaining a positive, pleasant, respectful, and socio-cultural relationship between the students themselves and with their teachers is the object of a good rapport (Budzi'nska and Majchrzak, 2021). The construction of rapport as a relationship variable has lately engrossed the thoughtfulness of linguists, educators, academics, scholars, and language teachers in the educational context (Catt et al., 2007; Frisby and Martin, 2010). Frisby et al. (2014) identified rapport as an educator tactic to lessen or even diminish intimidations when learners felt they were being exhibited through oral participation. They informed that the educator rapport was a face-caring method that condensed learners' face threat and fear to participate (Luo et al., 2020). The great rapport among learners and teachers has additionally been displayed to uniquely foresee learners' mentality toward the course and educator, enthusiasm, affective learners' education, and last course marks (Wilson and Ryan, 2013). According to Waples (2016), learners who witnessed good rapport with their educators could bear more difficult assignments inside a statistics course, assisting with working on efficacy and overcoming stress to succeed in the course. It was additionally inferred that learners are less reluctant to pose inquiries when they feel like they have a good connection with their educator.

This can be clarified by Frisby and Martin (2010), who discovered that rapport was associated with peer-to-peer connectedness, creating a more interesting class setting to which students could easily contribute. In this context, rapport has also been related to students' learning perceptions. Rapport predicts perception of cognitive learning, expected final grade, and actual final grades. Wilson and Ryan (2013) discovered that it was the engagement element of rapport, in specific, that anticipated these results (Frisby and Martin, 2010; Wilson et al., 2010; Frisby and Housley Gaffney, 2015). Moreover, Wilson and Ryan (2013) indicated that it was the commitment constituent of rapport that anticipated these results. A possible clarification for this is that education may be related to learners' propensity for pro-academic behavior, state motivation, and influence on the course when rapport with educators is positive (Sidelinger et al., 2015; Wang and Guan, 2020; Han and Wang, 2021). The effect on the teacher and learning associated with the perception of learning is also strongly related to rapport (Frisby and Housley Gaffney, 2015).

A measure of student stroke was developed and authenticated by Pishghadam and Khajavy (2014) to scrutinize the association between this issue, student stroke, and motivation, resulting in a constructive association between them. Similarly, a teacher stroke questionnaire was premeditated by Yazdanpour (2015) to inspect the association between teacher stroke and their burnout, indicating that the extent of stroke established by educators, either positively or negatively, would form their outlooks toward their occupations and their learners. Irajzad et al. (2017) led another research in which the strokes derived from high school learners in language courses in Iran were examined. The outcomes showed that educators give various kinds of strokes to students in these language developments. They predominantly ascribed their results to the kinds of structures they implement in their career. Pishghadam et al. (2019) studied the influence of teacher stroke and credibility on students' inclination to take part in classes. To this end, 276 learners freely participated in their research. Three valid and reliable scales were administered to measure the learners' perspectives toward the relations between the above-mentioned elements. The findings characterized those learners who supposed their teachers as well-informed and truthful were more motivated to be present in their classes. Educator stroke was also recognized as a motivational issue that improves learners' readiness and inclination to take part in classes.

## Methods

### Participants

Using the convenience sampling method, 316 participants were randomly selected online from more than 30 cities in nine provinces of China. All of them are Second Year (sophomore) non-English majors, closely connected with WeChat, and they were varied regarding gender, with 205 female students and 111 male students, and age, ranging from 18 to 21. Before participating in the survey, respondents that were certain their information would remain private and only used for educational determinations signed an agreement confirming their approval.

### Instruments

Based on the purposes of the study, these instruments were used as follows:

#### Grit Scale Questionnaire (Grit-S)

The researcher employed the Grit-S as a comparatively short Likert scale eight-item form containing two sections, that is to say, consistency of interest and perseverance of effort. This scale was established and authenticated by Duckworth and Quinn (2009) which is named the Grit-S where entries are graded and evaluated on a 5-point scale from 1 = not at all like me to 5 = very much like me to assess the level of grit in a person. Earlier studies (Duckworth and Quinn, 2009) recounted suitable to a good consistency for the Grit-S, through Cronbach α values with a range of 0.73–0.83.

#### Teacher Stroke Scale

Pishghadam and Khajavy (2014) used the learner Stroke Scale to evaluate strokes received by learners in the classroom. This scale consists of four underlying factors, namely verbal stroke (6 items: 5, 6, 7, 8, 13, 17), non-verbal stroke (4 items: 1, 2, 3, 4), valuing (4 items: 9, 10, 11, 12), and classroom activities (4 items: 14, 15, 16, 18). The scale is a 5-point Likert-type in which items range from 1 (never) to 5 (always); therefore, the participants' scores can range between 18 and 90. The reliability of the original scale was reported to be 0.88. Cronbach's alpha coefficient was also calculated for the present study which ranged from 0.79 to 0.88

#### Teacher-Student Rapport Scale

Originally, this scale was developed by learners based on items taken from their course assignments, which they claimed described their rapport with their educators (Wilson et al., 2010). A principal component analysis identified 34 items based on the evaluation of these items. These 34 items contained a distinct main issue, and all of its items got the lowest loading value of 0.50, with a reliability of 0.96. Each item was evaluated by attendees from extremely opposed to extremely agree on a scale of one to five. There were eight negative responses, while the other responses were positive (Wilson et al., 2010).

### Data Collection Procedures

In the present study, 316 male and female Chinese EFL participants within the age range of 18–21 took part in the online inspection. The three questionnaires, namely grit-s, teacher stroke scale, and teacher-student rapport scale, were sent to them through the WeChat application, due to barriers of the Covid-19, and they were asked to complete them and in case of any difficulties, they should be in contact with the researcher. It should be noted that participants were provided with abundant evidence on the progression of filling in the scales to accumulate the data. They were guaranteed that the information assembled by the surveys is solely for the goals of the study. To encourage them to answer the surveys, the analyst afforded them the chance to receive feedback on the results of each questionnaire by email. Statistical methods were directed in the last stage to assess the research hypotheses.

### Data Analysis

The study is a quantitative correlational research which is used to involve modeling and analyzing some variables. To this end, regression imputation was implemented in the Confirmatory factor analysis (CFA) model in the subsequent analysis. Indeed, regression analyses were used to check the predictor role of teacher stroke and teacher-student rapport on learners' grit.

## Results

### Pre-processing of the Data

Before starting the analysis, the data went through some pre-practice to eliminate the challenging data. Initially, 316 solid responses were obtained from the conduction of the questionnaires. No missing answer was found in the data, and the data were, first, inspected for patterns. Therefore, 28 cases with constant patterns, 3 cases with increasing patterns, and 1 case with decreasing patterns were identified and excluded. Then, the standard deviation of respondents' answers was calculated and those with values below 0.5 were inspected for non-engagement. Two such cases were found and excluded, leaving the final sample with 283 cases.

Initially, to verify the construct validity, a CFA was run. The initial model had three constructs (Grit, Stroke, and Rapport), each of which had some components. Grit, according to Teimouri et al. (2020) comprised of two components, namely, *effort* and *interest*. Teacher Stroke was made up of four components of *verbal, non-verbal, valuing*, and *classroom activities*, according to Pishghadam and Khajavy (2014). In analyzing the teacher-student rapport, we used the 15 reduced items extracted by Wilson and Ryan (2013) onto two components of *perception of teacher* and *student engagement*. First, each concept was probed for non-significant loadings in unstandardized valuation and/or low estimates (below 0.5) in standardized estimation. Table 1 shows the results.

**Table 1:** Unstandardized and standardize estimates of the initial CFA model.

			**Unstandardized**				**Standardized**
			**Estimate**	**S.E**.	**C.R**.	***P***	**Estimate**
G01	< —	Effort	1.000				0.769
G03	< —	Effort	1.083	0.080	13.520	0.000	0.783
G05	< —	Effort	1.098	0.077	14.309	0.000	0.824
G06	< —	Effort	1.082	0.075	14.363	0.000	0.826
G09	< —	Effort	0.672	0.079	8.463	0.000	0.514
G02	< —	Interest	1.000				0.306
G04	< —	Interest	1.674	0.375	4.464	0.000	0.502
G07	< —	Interest	2.015	0.436	4.621	0.000	0.567
G08	< —	Interest	1.084	0.293	3.697	0.000	0.501
S05	< —	Verbal	1.000				0.726
S06	< —	Verbal	1.023	0.081	12.597	0.000	0.758
S07	< —	Verbal	0.983	0.089	11.004	0.000	0.666
S08	< —	Verbal	−0.180	0.061	−2.938	0.003	−0.181
S13	< —	Verbal	1.107	0.084	13.116	0.000	0.788
S17	< —	Verbal	0.954	0.082	11.611	0.000	0.701
S01	< —	Non-Verbal	1.000				0.538
S02	< —	Non-Verbal	0.180	0.089	2.011	0.044	0.125
S03	< —	Non-Verbal	0.975	0.129	7.571	0.000	0.566
S04	< —	Non-Verbal	1.206	0.137	8.810	0.000	0.722
S09	< —	Valuing	1.000				0.504
S10	< —	Valuing	0.873	0.147	5.949	0.000	0.427
S11	< —	Valuing	1.359	0.197	6.910	0.000	0.530
S12	< —	Valuing	1.443	0.201	7.187	0.000	0.564
S14	< —	Activities	1.000				0.745
S15	< —	Activities	1.055	0.076	13.924	0.000	0.807
S16	< —	Activities	1.059	0.076	13.989	0.000	0.810
S18	< —	Activities	0.174	0.065	2.658	0.008	0.163
R11	< —	Engagement	1.000				0.716
R14	< —	Engagement	0.892	0.105	8.523	0.000	0.511
R15	< —	Engagement	1.200	0.104	11.516	0.000	0.686
R16	< —	Engagement	0.849	0.128	6.608	0.000	0.397
R17	< —	Engagement	0.804	0.099	8.131	0.000	0.487
R19	< —	Engagement	1.053	0.114	9.246	0.000	0.553
R26	< —	Perceptions	1.095	0.060	18.296	0.000	0.887
R27	< —	Perceptions	1.127	0.058	19.499	0.000	0.924
R28	< —	Perceptions	1.130	0.056	20.114	0.000	0.941
R29	< —	Perceptions	1.103	0.054	20.313	0.000	0.947
R30	< —	Perceptions	1.108	0.066	16.865	0.000	0.840
R22	< —	Perceptions	1.000				0.804
R25	< —	Perceptions	1.078	0.066	16.262	0.000	0.820
R32	< —	Perceptions	1.077	0.056	19.327	0.000	0.919
R31	< —	Perceptions	1.069	0.056	18.990	0.000	0.909

**Figure 1:**
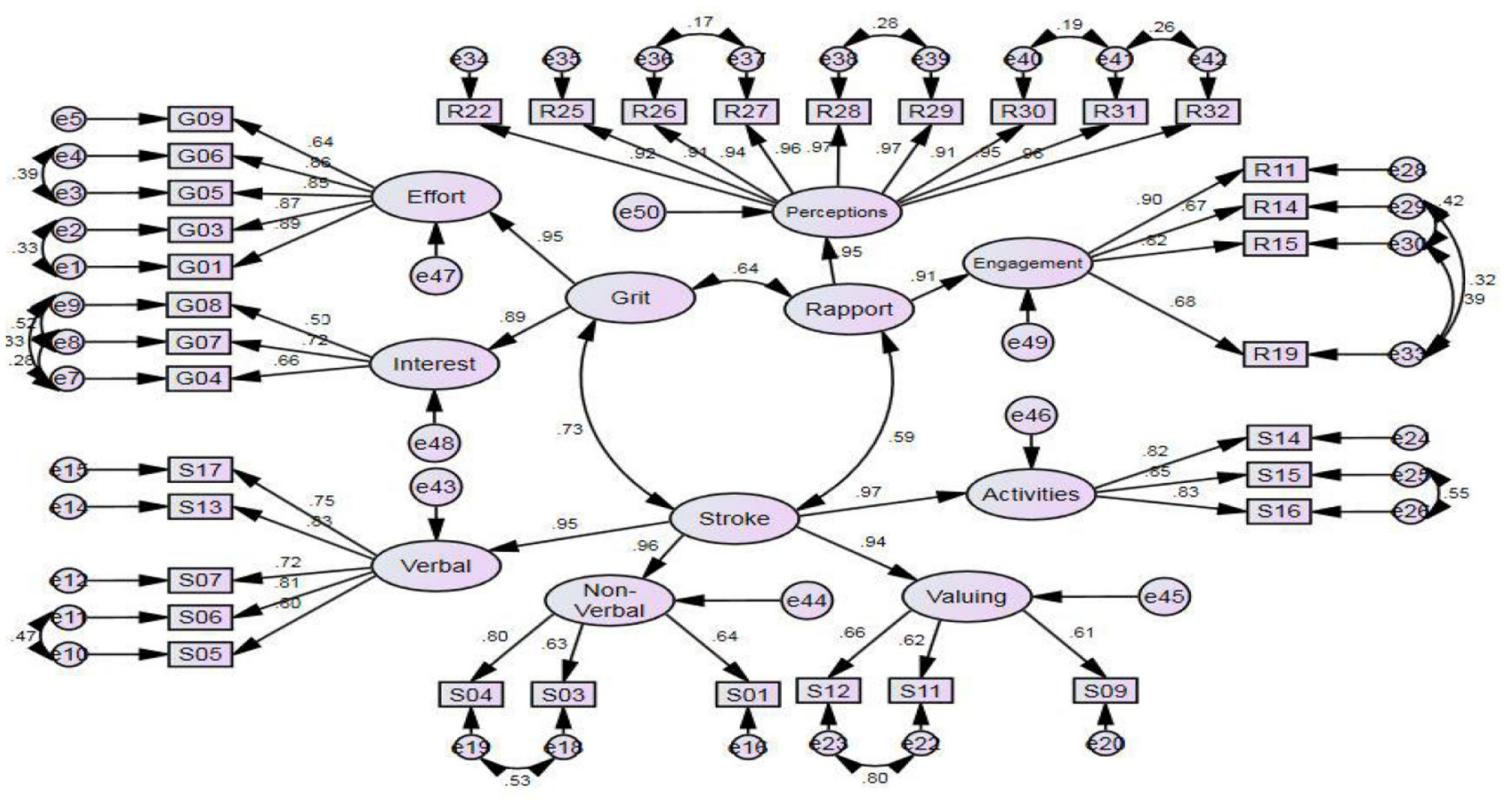
The final modified CFA model with standardized estimates.

As reported, no non-significant unstandardized approximations were established. However, 10 items, i.e., 1 (G02) from Grit, 4 (S02, S08, S10, and S18) teacher stroke, and 2 (R16 and R17) from teacher-student rapport had standardized estimates below 0.45, thus, they were excluded. Next, the adjustment indices with the inception of 10 were checked and the recommendations that were not contradictory to the literature were sought. Figure 1 delineates the ultimate adapted CFA model.

After applying the modifications, the model's goodness of fit was examined. As stated by Hu and Bentler (1999), for the model to have a goodness of fit, several criteria have to be met. These criteria, alongside the values obtained from the data, are testified in Table 2.

**Table 2:** Evaluation of the CFA goodness of fit.

**Criteria**		**Threshold**			**Evaluation**
		**Terrible**	**Acceptable**	**Excellent**	
CMIN	1222.253				
df	540				
CMIN/df	2.263	>5	>3	>1	Excellent
RMSEA	0.067	>0.08	<0.08	<0.06	Acceptable
CFI	0.913	<0.9	>0.9	>0.95	Acceptable
TLI	0.905	<0.9	>0.9	>0.95	Acceptable
SRMR	0.078	>0.1	>0.08	<0.08	Excellent

The results reported in Table 2 indicate satisfactory to outstanding goodness of fit. Next, the composite reliability (CR) and discriminant validity for each aspect was inspected (Table 3). As reported, all of the factors had CR values above 0.7, which reveals acceptable reliability. The average variance explained for each factor was safely exceeding 0.5 and the maximum shared variance (MSV) for each factor was below its average variance extracted (AVE). These two conditions support the convergent validity of the model. Moreover, the square root of AVE (the bold values in the table) was above inter-correlations of the factors, indicating discriminant validity, according to Fornell and Larcker (1981).

**Table 3:** Reliability and validity of the factors.

				**Fornell—Larcker Criterion**		
	**CR**	**AVE**	**MSV**	**Grit**	**Stroke**	**Rapport**
Grit	0.922	0.855	0.535	0.924		
Stroke	0.977	0.914	0.535	0.731**	0.956	
Rapport	0.932	0.872	0.409	0.640**	0.592**	0.934

**Correlation is significant at p < 0.01.

The inspection of the correlations (values not in bold under Fornell-Larcker Criterion) acknowledged that there are consequential correlations between all couples of elements. Strong correlations were found between teacher stroke and grit (*r* = 0.731), teacher stroke and teacher-student rapport (*r* = 0.592), and grit and teacher-student rapport (*r* = 0.640). Using regression imputation, the data in the CFA model was imputed to be used in the subsequent analysis. Regression imputation works like calculation of the average scores for each component, yet it is a more accurate measure as it takes into account the weighted share of each item in calculating the average. In other words, each item is weighted based on its share of explaining the average variance of its component. Table 4 presents the descriptive statistics for the imputed constructs.

**Table 4:** Descriptive statistics of the scores after regression imputation (*N* = 282).

		**N**	**Min**.	**Max**.	**Mean**	**SD**	**Skewness**	**Kurtosis**
Grit	Effort	464	1.23	5.56	3.5785	0.73631	0.205	0.523
	Interest	826	0.68	3.62	2.3989	0.47872	0.038	0.451
	Total	1,290	1.32	5.48	3.5893	0.67888	0.192	0.528
Teacher stroke	Verbal	464	1.14	5.18	2.8800	0.80274	0.538	0.444
	Non-verbal	826	0.88	3.84	2.3032	0.55309	0.379	0.418
	Valuing		0.76	3.31	1.9871	0.47477	0.400	0.390
	Activities		1.25	5.46	3.1517	0.83902	0.454	0.312
	Total	1,290	1.22	5.16	3.0334	0.75188	0.463	0.426
Teacher-student rapport	Engagement	464	2.30	4.57	3.9648	0.53067	−0.711	−0.353
	Perceptions	826	3.00	5.63	4.9306	0.70548	−0.697	−0.577
	Total	1,290	3.12	5.68	4.8581	0.65196	−0.627	−0.616

**Figure 2:**
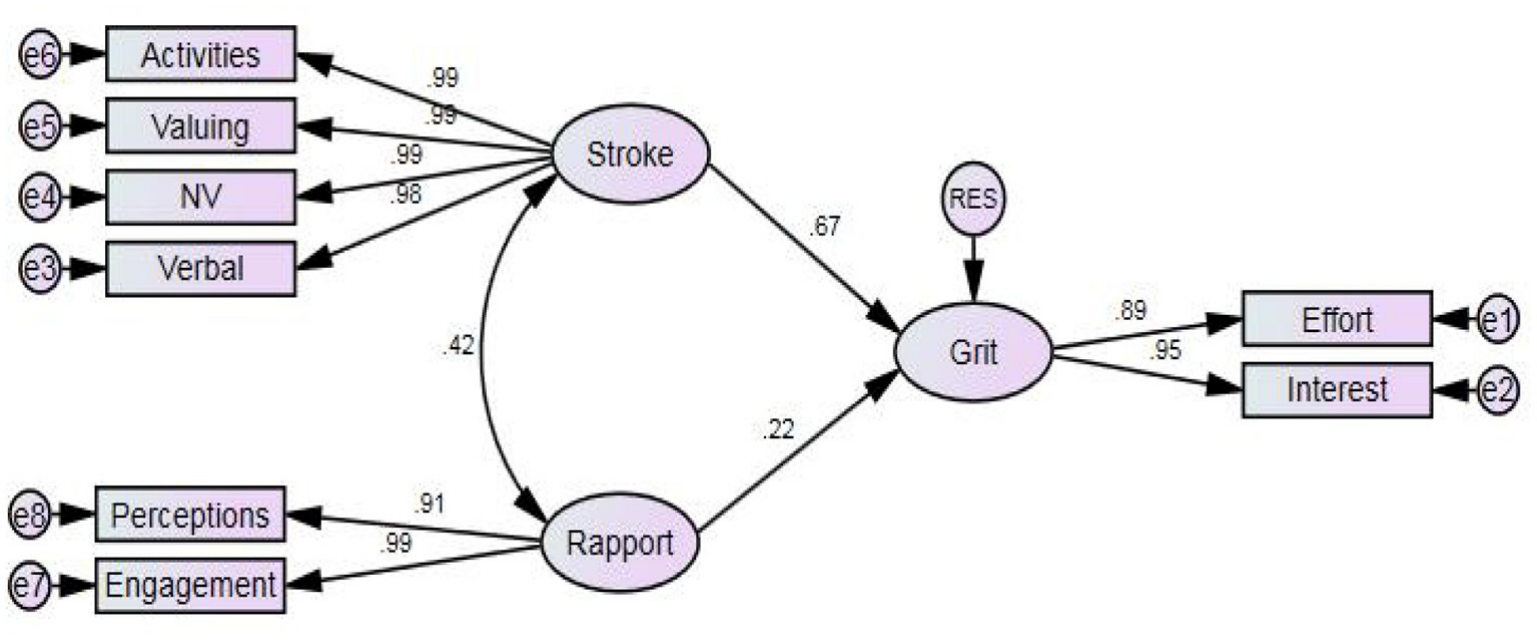
The measurement model.

As reported in Table 4, all distributions of scores showed normalcy as both skewness and kurtosis values were lower than its total value of 3. A regression analysis through employing structural equation modeling was run to observe the predictability grit by teacher-student rapport and teacher stroke. The measurement model is presented in Figure 2.

Table 5 reports the results of the analysis. It should be noted that, in running the analysis, the imputed values extracted from CFA were used. As reported in Table 5, after taking into account the covariance between teacher stroke and teacher-student rapport, the two variables could jointly predict 62.4% of the variance in grit. Both variables were the significant predictor of grit, while teacher stroke was better predictor (β = 0.675, *p* = 0.000 < 0.01), uniquely explaining 45.5% of the grit's variance. Teacher-student rapport was also proved to be a predictor of grit (β = 0.215, *p* = 0.000 < 0.01), uniquely explaining 4.6% of its variance.

**Table 5:** Results of multiple linear regression analysis with SEM.

			**Weight**	**S.E**.	**C.R**.	***P***	**β**	***R* ^2^**	**Multiple correlation**
									***R* ^2^**
Grit	< —	Stroke	0.442	0.033	13.519	0.000	0.675	0.455	0.624
Grit	< —	Rapport	0.211	0.046	4.576	0.000	0.215	0.046	
Stroke	< –>	Rapport	0.174	0.027	6.435	0.000	0.421		

## Discussion

The current investigation tries to scrutinize the influences of teacher stroke and teacher-student rapport on students' grit in language learning in the Chinese EFL milieu. Given the predictive relationship between teacher rapport and learners' grit, it is revealed that practitioners can help teachers and students increase their relationship and interaction, which in turn might enhance the learners' grit. Undoubtedly, The upshots of this learning support the past investigations on rapport structure, by displaying that there is an apparent association between teacher-student relationship and students' grit. Interpersonal communication is dynamic in the learning process and making connections in this regard has been presented to be an actual method of collaborating with learners. By involving in surprisingly observant performances, involving activities, information sharing manners, considerate performances, and shared basis manners, teachers will confidently experience developed teacher and student relations, which bring about more operational education, and, eventually, develop learners' grit that leads to their attentiveness, interests, enthusiasm and finally their learning success (Webb and Barrett, 2014).

Likewise, when teachers cooperate with students and have a respectful and compassionate outlook toward students, their anxiety and tensions reduce and they can wholly emphasize their educations with the great inspiration and enthusiasm that is consistent with Mercer and Gkonou (2020), who demonstrated that the L2 Motivational Self System generates a wonderful course of attention and accomplishment and thus, it can be definite that individuals with a great level of grit are thought to be proficient of cumulative their abilities since they have more consideration and are less disappointed by difficulties, challenges and problems (Credé et al., 2017).

Another important reasoning of the present study is that rapport building can have constructive effects in the teaching space (Coupland, 2003) as it can minimize stress, increase student engagement, form and encourage social association, promote a positive educational setting, and increase learning. Greater interaction and participatory activities also enhance the relationship between educators and learners. The educator must create circumstances so the whole class is guaranteed a place and time to interact regularly with the educator on various topics and issues relevant to the class. A significant part of education and learning is sustaining good rapport with learners in and out of class and educational premises that assists the teacher with maintaining a genuine, responsible, and motivated class (Pishghadam and Khajavy, 2014), thus, the teacher must sustain it. As teacher-learner interactions bring about a creative classroom atmosphere and individual learner success, educators should be cognizant that stroking should be an integral section of their job and can be a useful technique for managing the class (Havik and Westergård, 2020). In this sense, teacher instructors are required to create better connections with pre-service educators, primarily as a result of the point that teachers' comprehension of the notion of educator stroke is strongly impacted by their correlation with teacher instructors.

The importance of educator stroke has been emphasized when it comes to understanding how constructive learner-educator relational connections can have a constructive effect on learners' education, inspiration, self-esteem, the satisfaction of learners' hunger for emotive maintenance, and their yearning for enjoyable states of education, among other advantages (Pekrun and Schutz, 2007). The findings indicate that more teacher-student communication is concerning learners' grit that is in agreement with the study done by Peng and Woodrow (2010) who stated that successful teacher-student communication can boost learners' enthusiasm, the accomplishment of crucial relational skills, and commitment, along with reduction of their anxiety level that it leads to more grit in learners.

Consequently, by implementing interactional behaviors, and collaborating with students both in the class and before and after the class, teachers are intended to achieve higher motivation leading to instructive and emotive results. As stated by Xie and Derakhshan (2021), teacher precaution, intelligibility, trustworthiness, relationship with students, stroke, immediacy, and validation develop in what way educators anticipate the educational consequences such as enthusiasm, commitment, class participation, presentation, and achievement in learners. As a result, the stated aspects in educators can be regarded as constructive which causes learners to feel pleased and let their desires be fulfilled (Goldman et al., 2017; Wang and Guan, 2020).

The outcomes of this inquiry support the one carried out by Rajabnejad et al. (2017) who have tried to study the function of teacher stroke in improving learners' willingness to take part in courses among Iranian EFL learners. The findings of their research indicated that Learners who attained strokes from their educators showed greater inclination to go to class, and thus educators' stroke was recognized as a key indicator for learners' willingness to go to class. In this way, learners can better commit to long-standing tasks, determine resilience throughout challenging circumstances and display more achievement in all aspects of life (Duckworth, 2016).

## Conclusion and Implications

The present study could have particular suggestions for investigators, educators, and teacher instructors in the framework of English as a foreign language. From an operational point of view, the development of the interrelationships between the characteristics of educators and learners in the medium of EFL can be important for the development of students' grit. Also, this study can help the language educator to find ways to enhance students' grit. A successful foreign language education program can depend in great part on the rapport between instructors and students. Several studies have shown that a positive learning atmosphere can be achieved through building rapport. A connection-based term such as rapport helps describe how people perceive each other. As an insightful review, the following paper emphasizes the significance of learner-educator rapport, which encourages instructors to set and design new teaching strategies and improve their ideas about how to build meaningful connections that motivate learners toward learning and success. Educator trainers could regard the findings of this research as interesting, as well. Educator training courses must attend to the notions of stroke and rapport as a means to elevate learners' grit.

The findings of this research can be instructive for language educators. Focusing on learners and encouraging them to participate in classroom debates and activities not only assists in increasing learners' motivation but also, as shown in the results of the current research, has a significant part in students' grit. It is consequently suggested that educators try to offer learners a stroke-rich setting and be conscious about the deconstructive impacts of under-stroking on students' grit. Consequently, teachers are acclaimed to make an effort to make a stroke-rich atmosphere for their learners and to be conscious of the destructive influences of this type of circumstances on the students' grit that is an important aspect of their success. Furthermore, to be regarded as credible in the eyes of their learners, educators need to put effort for the sake of being more successful and impactful in their work. An educator must be perceived as a subject matter professional who cares for and comprehends learners, and thus someone the learners can have faith in that can trigger their grit level.

Through interaction, students can develop persistence, as it is significant that students have a high level of perseverance and effort to meet their educational needs. Moreover, educators should offer emotive provision by classifying students' efforts to actively participate in various assignments. Through association between teachers and students, students have been provided chances to tackle hard tasks, care for and motivate one another, create a collaborative environment, and improve their grit level. By providing the opportunity to pursue longstanding goals worthy of students' resilience and by providing a challenging and compassionate environment to achieve their goals, higher academic managers can enhance student achievement. Educators and experts could emphasize the achievement of relationships in the development of education by promoting compassionate and cooperative assignments and activities that allow group collaborative learning to aid them in the learning process, improve their grit level, and upsurge their scholastic performance, determination, and engagement (Lan and Moscardino, 2019; Xie and Derakhshan, 2021). In addition, positive relationships between educators and learners lead to a classroom climate that promotes instruction and turns into the learners' behavioral and academic demands. An effective change in the educational environment of learners occurs when a learner-educator rapport is established at the beginning of the formal education process.

Textbook designers should know that the more realistic and communicative a coursebook is, the more communication there is in the teaching space and the more strokes are given. As a result, they need to enhance the construction of the textbooks to allow for elasticity in teachers. Furthermore, curriculum designers should design activities in the classroom to allow teacher-student communication, as it was a guide for them to share information with their classmates and their educators who fostered their efforts.

Teacher trainers must change educators' mentalities toward and awareness of their instructional obligations. In other words, they must make educators conscious of the fact that their obligations in educational learning settings go beyond the relay of material and educational information. Thus, educators should know that the relational practices they execute in their association with learners are just as significant as their information and educational abilities.

Moreover, educational supervisors with the obligation of observing educators and assessing their scholastic effectiveness can make use of the study evidence in the domain of constructive educator-learner connections. Aside from educator capacity and educational abilities, supervisors need to consider relational practices as well due to the central part of educators' relational practices in the sufficiency of education and learning. By offering classes, conferences, and other teaching dimensions relevant to educator-learner rapport and stroke, educator trainers can motivate new teachers to develop in their comprehension of the scholastic, relational, and mental aspects of education.

Research like this suggests new teaching methods and stimulates the educator to reconsider his or her view of learners and determine how to build a rapport with them that encourages grit and self-confidence. Most studies on educator-learner rapport and positive learner results include a correlational research format. To support a causal connection, future studies should utilize experimental formats with controls of factors straightforwardly identified with rapport and resulting measures of learners' education. This research was carried out in colleges in China. Future researches can investigate the factors present in this research within other contexts, such as public schools or language organizations, to witness potential variations in the outcomes. Furthermore, any generalizations about the findings should be made with caution since the data sources of this research were English language students in only 30 cities in China, and thus the results cannot be indicative of all EFL learners in China. Moreover, since the present study obtained data about educators from the standpoint of their learners, future studies can use educators' views about themselves in addition to educators' observations to triangulate the information. It is recommended that future studies take into account individual differences among educators like gender or years of experience.

## Data Availability Statement

The original contributions presented in the study are included in the article/supplementary material, further inquiries can be directed to the corresponding author/s.

## Ethics Statement

The studies involving human participants were reviewed and approved by Xinxiang Medical University Research Ethcis Committee. The patients/participants provided their written informed consent to participate in this study.

## Author Contributions

LY conceptualized, designed the current study, collected the data, and drafted the first manuscript.

## Conflict of Interest

The author declares that the research was conducted in the absence of any commercial or financial relationships that could be construed as a potential conflict of interest.

## Publisher's Note

All claims expressed in this article are solely those of the authors and do not necessarily represent those of their affiliated organizations, or those of the publisher, the editors and the reviewers. Any product that may be evaluated in this article, or claim that may be made by its manufacturer, is not guaranteed or endorsed by the publisher.
